# A Complex Case of Dual Ocular Pathologies in an Adolescent: A Diagnostic Challenge

**DOI:** 10.7759/cureus.90202

**Published:** 2025-08-16

**Authors:** Syamin Mohd Noor, Aidila Jesmin Jabbari, Abdul-Hadi Rosli

**Affiliations:** 1 Ophthalmology, Kulliyyah of Medicine, International Islamic University Malaysia, Kuantan, MYS

**Keywords:** bartonella henselae, cat scratch disease, ocular bartonellosis, vkh, vogt-koyanagi-harada

## Abstract

Clinical manifestations of ocular disease may overlap, and although rare, patients may suffer from more than one disease consecutively, complicating the diagnostic process. This case highlights an uncommon presentation of Vogt-Koyanagi-Harada (VKH) disease following *Bartonella henselae* infection in a 15-year-old girl.

She presented with a two-month history of bilateral eye blurring of vision, associated with a throbbing headache. Examination revealed the presence of anterior uveitis with bilateral optic disc swelling and partial macular star. Diagnosis of bilateral ocular bartonellosis with neuroretinitis was made based on positive serology. Following treatment of oral azithromycin for six months, her vision improved with resolution of neuroretinitis.

Eleven months later, she presented again with blurred vision in both eyes. Fundus examination revealed bilateral optic disc swelling and retinal pigmentary changes with right exudative macular detachment. The investigations were negative for an infective or inflammatory origin. Fundus fluorescein angiography (FFA) showed minimal pinpoint leakage in the early phase and a moth-eaten appearance in the late phase. These findings led to the diagnosis of chronic Vogt-Koyanagi-Harada (VKH) disease. This case emphasizes the importance of having a high degree of suspicion to ensure correct diagnosis and management of patients.

## Introduction

Ocular bartonellosis is an ocular involvement of cat scratch disease caused by the gram-negative bacillus *Bartonella henselae*. The bacteria are transmitted to humans by cat bites, licks, or scratches. Primary inoculation often causes systemic reactions, such as fever, malaise, and regional lymphadenopathy. Typical intraocular presentation includes Parinaud oculoglandular syndrome, follicular conjunctivitis, and neuroretinitis. Neuroretinitis, which occurs in 1%-2% of patients [1], is characterized by optic disc swelling with macular star formation and discrete white retinal or choroidal lesions. Clinically, infected patients commonly experience unilateral reduction in vision, scotoma, and occasional flares and uveitis. A positive serology for *Bartonella henselae* confirms the diagnosis, and initiation of antibiotics such as oral azithromycin will effectively treat the infection, resulting in symptom resolution within a few weeks.

Vogt-Koyanagi-Harada (VKH) disease is an idiopathic multisystem autoimmune disorder featuring inflammation of the melanocyte-containing tissue, such as the uvea, ear, and meninges. It predominantly affects heavily pigmented individuals, particularly Hispanic, Asian, and Native American descendants. VKH often manifests as bilateral granulomatous panuveitis and is associated with serous retinal detachment, vitritis, disc swelling, pigmentary changes, scarring, and eventual development of sunset glow fundus. Systemically, it is associated with central nervous system involvement such as meningeal irritation or encephalopathy, with other cutaneous and auditory manifestations such as deafness, tinnitus, vitiligo, and poliosis.

Diagnosis is based on specific criteria, which include bilateral uveitis with absence of other ocular disease, no history of ocular trauma or surgery, presence of neurological and auditory manifestations, and integumentary findings such as alopecia, poliosis, and vitiligo. Prompt diagnosis and early initiation of high-dose steroids in controlling the inflammation are crucial to prevent vision loss.

The aim of this case report is to describe an uncommon case of VKH disease following *Bartonella henselae* infection in a young adult and to highlight the challenges and clinical reasoning in diagnosing VKH in this patient.

## Case presentation

**Figure 1:**
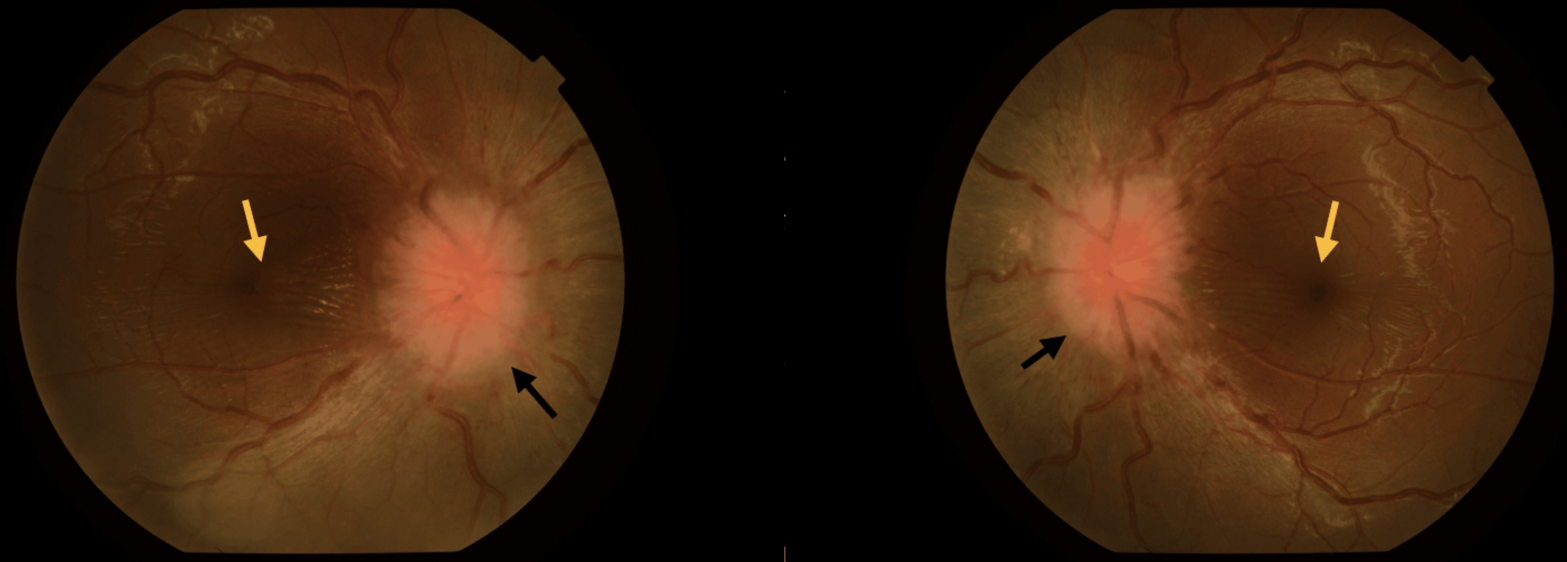
Bilateral optic disc swelling (black arrow) with partial macular star (yellow arrow)

A 15-year-old Malay girl presented with a two-month history of bilateral eye blurring of vision, associated with throbbing headache and eye discomfort. General examinations showed that she was afebrile with stable vital signs. Best corrected visual acuity (BCVA) of the right eye (RE) was 6/18 and the left eye (LE) was 6/15. Examination revealed the presence of anterior uveitis with multiple areas of posterior synechiae, as well as vitritis in both eyes. Intraocular pressure and anterior chamber angle were normal. Fundus examination showed bilateral neuroretinitis, evidenced by the presence of optic disc swelling and partial macular star (Figure 1).

Infectious screening, including *Toxoplasma gondii*, rubella virus, cytomegalovirus, herpes simplex virus, and syphilis (TORCHES), Venereal Disease Research Laboratory (VDRL), and QuantiFERON-tuberculosis (TB), were negative. However, her erythrocyte sedimentation rate (ESR) was raised (51 mm/hour). Computed tomography (CT)of the brain and orbit was normal. A positive *Bartonella henselae* serology for both immunoglobulin M (IgM) (titer: 1:20) and immunoglobulin G (IgG) (titer: 1:128) confirmed the diagnosis of bilateral neuroretinitis secondary to ocular bartonellosis. She was started on oral azithromycin 500 mg daily for six weeks and topical dexamethasone 0.1% four hourly, which was then tapered down within 10 weeks. Following treatment, there was resolution of the neuroretinitis and improvement in her symptoms. Her BCVA improved to 6/9 for the right eye and 6/6 for the left eye.

Eleven months later, the patient presented with a new onset of reduced vision in both eyes. BCVA of RE was 6/18 and LE was 6/15. She denied any headache, skin lesions, or ear symptoms. Anterior segment examination revealed anterior uveitis with posterior synechiae in bilateral eyes. Fundus examination revealed bilateral optic disc swelling with the presence of Dalen-Fuchs nodules over the peripheral retina.

**Figure 2:**
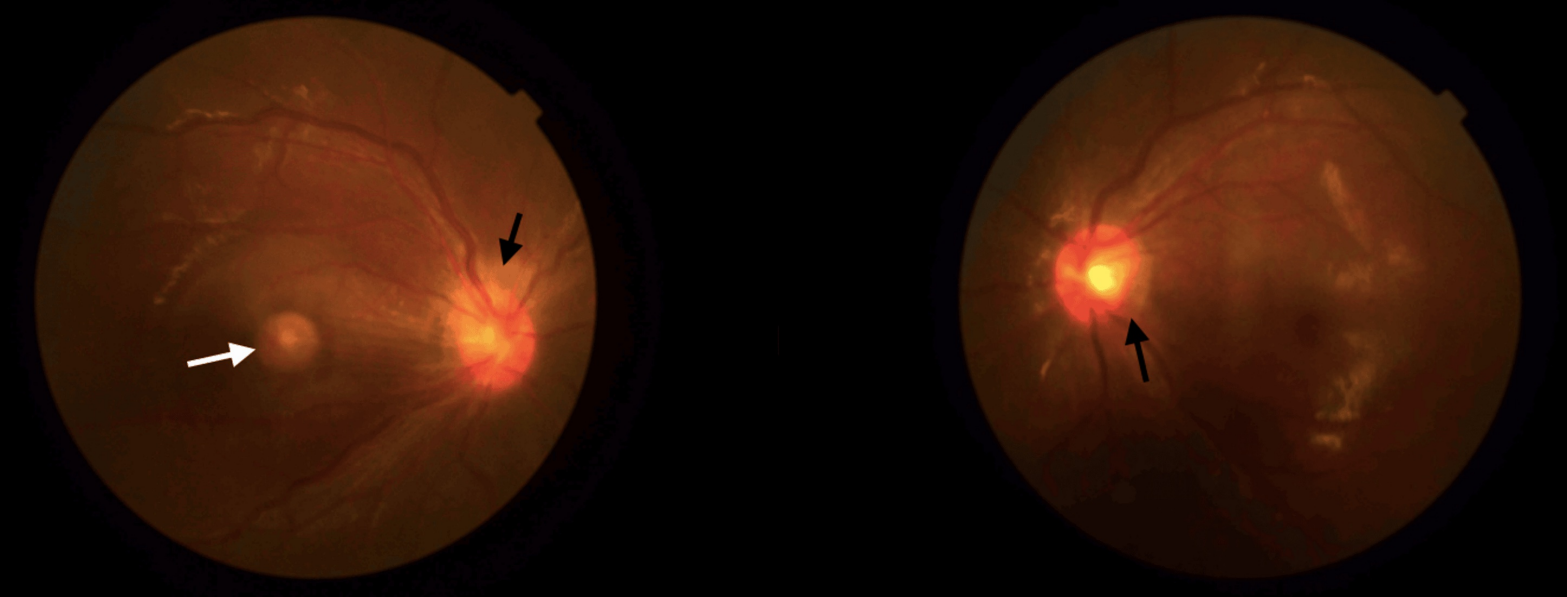
Bilateral optic disc swelling (black arrow) with exudative macular detachment over the right eye (white arrow)

The repeated investigations were negative for an infective or inflammatory origin. Since the initial diagnosis of ocular bartonellosis was supported with positive serology and good response to treatment, the patient was treated as a recurrence or reactivation of ocular bartonellosis. Consequently, a six-week course of oral azithromycin 500 mg daily was initiated. However, subsequent follow-up revealed worsening symptoms, including the development of a central scotoma in the right eye despite ongoing treatment. BCVA of RE worsened to light perception (LP), and LE BCVA was 6/15. Fundus examinations revealed bilateral optic disc swelling and right choroidal granuloma and exudative macular detachment (Figure 2).

**Figure 3:**
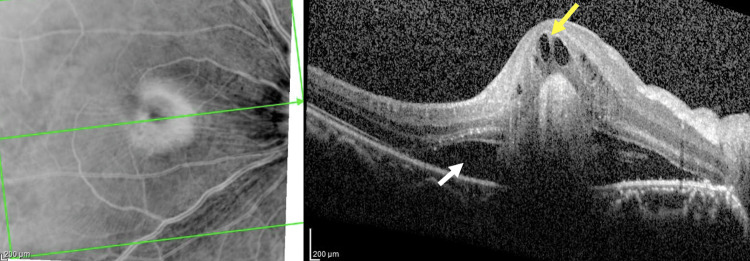
OCT of the macula of the right eye showing right neurosensory detachment with subretinal fluid (white arrow) and multiple intraretinal septae (yellow arrow) OCT: optical coherence tomography

Optical coherence tomography (OCT) showed right serous retinal detachment as evidenced by neurosensory detachment with cystoid macula edema and multiple septae (Figure 3).

**Figure 4:**
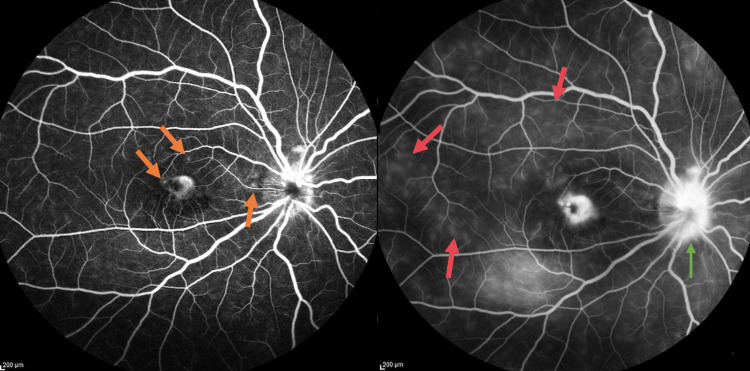
Sequential fundus fluorescein angiogram in the right eye showing pinpoint leakage (orange arrow) in the early phase and a moth-eaten appearance (red arrow) in the late phase with hot disc (green arrow)

Fundus fluorescein angiography (FFA) later showed minimal pinpoint leakage in the early phase and a moth-eaten appearance in the late phase with hot disc (Figure 4). These findings led to the diagnosis of chronic Vogt-Koyanagi-Harada (VKH) disease.

She was started on systemic prednisolone 1 mg/kg daily for six weeks. Following treatment, her vision improved with BCVA of 2/120 for the right eye and 6/6 for the left eye. However, the vision of the right eye remained poor due to the presence of a macular scar. The patient defaulted on subsequent follow-up appointments, hence limiting the opportunity to provide visual rehabilitation support.

## Discussion

This case highlights the complexity of diagnosing two distinct ocular conditions that occur simultaneously in a young patient. She initially presented with ocular bartonellosis and later developed features and signs consistent with VKH. The clinical presentation of ocular diseases can overlap, causing a diagnostic challenge, especially as VKH is rarely seen after *Bartonella* infection.

Ocular bartonellosis can manifest as neuroretinitis, uveitis, retinitis, chorioretinitis, vasculitis, retinal vascular occlusions, and orbital abscess [2,3]. Neuroretinitis is usually unilateral, but bilateral involvement occurs in approximately 2% of cases [2]. To the best of our knowledge, there is limited literature reporting cases of bilateral neuroretinitis in children [4]. The diagnosis of *Bartonella henselae *infection is confirmed by serologic testing [5]. Antimicrobial therapy using antibiotics such as doxycycline and macrolides has been shown to be effective against the organism [6]. In this case, the patient showed significant improvement in vision with resolution of neuroretinitis after initiation of treatment.

Challenges arise when the patient returned with a new complaint of bilateral eye blurred vision. In this case, the preceding ocular *Bartonella* neuroretinitis leads to diagnostic ambiguity when the patient came for the second presentation. Previous reported cases of recurrent ocular bartonellosis [7,8] and a similar case of bilateral neuroretinitis with exudative retinal detachment secondary to *Bartonella henselae* infection documented by Fleissig et al. [9] lead toward the diagnosis of ocular bartonellosis. However, the patient's worsening condition despite antibiotics raised suspicion of an alternative pathology. Diagnosis of VKH was later established after fundus fluorescein angiography demonstrated the presence of minimal pinpoint leakage and a moth-eaten appearance. Although rare, a similar case of VKH disease following *Bartonella henselae* retinitis has been reported in 2022 [10].

VKH typically progresses through four phases: prodromal, acute uveitic, convalescent, and chronic recurrent stage. The prodromal phase manifests as a non-specific viral-like illness, including headache, fever, nausea, and dizziness. However, these were absent in our patient. Following this, the acute uveitic phase is characterized by panuveitis, diffuse choroidal infiltration, optic disc swelling, and serous retinal detachment. Dalen-Fuchs nodules may also be observed as a hallmark of granulomatous inflammation. Depigmentation, such as vitiligo, poliosis, and sunset glow fundus, is the characteristic feature of the convalescent phase [11]. Recurrent panuveitis with anterior uveitis is observed in the chronic recurrent stage, often with the presence of vision-threatening complications such as glaucoma, cataract, and subretinal neovascularization. Clinical manifestations of VKH vary according to the stages of disease; however, patients may not necessarily manifest all the signs of each stage, causing difficulties in establishing the diagnosis and hence treatment delay. Studies revealed that 9.2% of VKH cases are misdiagnosed at initial presentation [12].

Multimodal imaging, such as FFA and indocyanine green angiography (ICGA), offers supportive evidence in diagnosing early and atypical presentations, as well as excluding conditions mimicking VKH [13]. In this case, FFA was key to the diagnosis, although our setting lacked ICGA, which would demonstrate typical hypofluorescent dark patches corresponding to exudative retinal detachment. OCT is a valuable non-invasive imaging in evaluating disease staging and monitoring treatment efficacy. In the acute phase, OCT showed exudative retinal detachment, disorganized inner retinal layers, thickened choroid, fibrinous septa in the subretinal space, and retinal folds without retinal pigment epithelium (RPE) detachments [14]. In this case, OCT confirms the presence of serous retinal detachment and choroidal thickening, which is the hallmark of active VKH. The lack of a single definitive test in VKH further complicates reaching the diagnosis. The diagnosis of VKH relies heavily on clinical judgement, with support from multiple imaging and exclusion of other causes.

## Conclusions

The occurrence of two distinct ocular pathologies in a single patient, with atypical presentation, requires careful consideration of the differential diagnosis. Continuous monitoring and evaluation of the patient's progress may offer insights into the exact diagnosis. A high degree of suspicion and reassessments when patient conditions worsen are key to a correct diagnosis. Early management is vital for better visual outcomes and prevents further complications that are irreversible with poor visual prognosis.
